# Human testis-expressed sequence 101 is limitedly distributed in germinal epithelium of testis and disappears in seminoma

**DOI:** 10.1186/0717-6287-47-52

**Published:** 2014-10-01

**Authors:** Cong-Cong Shen, Yu-Huan Kang, Lin Yu, Dan-Dan Cui, Yi He, Jin-Liang Yang, Lan-Tu Gou

**Affiliations:** State Key Laboratory of Biotherapy/Collaborative Innovation Center for Biotherapy, West China Hospital, Sichuan University, No. 1, Keyuan Road 4, Gaopeng Street, Chengdu, 610041 PR China; Department of Medical Oncology, the Fifth People’s Hospital of Chengdu, Chengdu, 611130 Sichuan Province PR China

**Keywords:** Immunohistochemistry, TEX101, Seminoma, Testis, Western blotting

## Abstract

**Background:**

Testis-expressed sequence 101 (TEX101) was found to be highly expressed in testis and involved in acrosome reaction in previous studies. Recently, the metastasis suppressor function of TEX101 in cancer was disclosed, but the comprehensive investigation of its expression has rarely been reported. In this study, the expression features of TEX101 in normal human organs and seminoma were systematically analyzed.

**Results:**

Immunohistochemistry demonstrated intense staining of TEX101 in human testis tissues; however, its expression in 27 other types of normal human organs, including the ovary, was negligible. Higher expression of TEX101 was observed in the spermatocytes and spermatids of the testis, but relatively lower staining was detected in spermatogonia. Western blotting showed a single TEX101 band of 38 kDa in human testis, but it did not correspond to the predicted molecular weight of its mature form at 21 KDa. Furthermore, we examined seminoma tissues by immunohistochemistry and found that none of the 36 samples expressed TEX101.

**Conclusions:**

Our data confirmed TEX101 to be a testis protein that could be related to the maturation process of male germ cells. The lack of TEX101 in seminoma indicated its potential role in tumor progression. This characteristic expression of TEX101 could provide a valuable reference for understanding its biological functions.

## Background

Human testis-expressed sequence 101 (TEX101), also known as testis-expressed sequence 101, is a recently identified protein. The TEX101 gene is located on human chromosome 19q13.2 and encodes 249 amino acids including a secretion signal peptide (1–25 amino acids) at its N-terminus. After the signal peptide and its C-terminus (223–249 amino acids) are removed, TEX101 is transformed into its mature form, presenting a molecular weight of 21 kDa and an isoelectric point of 4.7. Bioinformatics analysis shows that TEX101 is a membrane GPI-anchored protein with a conserved UPAR/Ly6 domain, indicating a similar protein structure as urokinase-type plasminogen activator receptor (uPAR) [1].

TEX101 was initially identified in mice and showed a limited distribution with high expression in testis [2–4]. Subsequently, TEX101 in human testis was identified using cDNA microarray [5] and found to be involved in the acrosome reaction in the process of insemination [6]. The acrosome reaction occurs in the acrosome of the sperm when it interacts with the egg and is important for mammalian fertilization [7, 8]. Because of its specific significance in testis, TEX101 has been utilized as a biomarker for male infertility, and this was confirmed to diagnose azoospermia by means of testicular biopsy by Drabovich’s team [9]. More recently, the expression and behavior of TEX101 in cancer were also investigated and showed potential significance for cancer progression [10–12]. YIN et al. found that TEX101 could bind to uPA/uPAR complexes and interfere with the activities of uPA, matrix metalloproteinases and cathepsin B, which resulted in the reduction of extracellular matrix degradation and consequent suppression of cancer invasion [13].

The emerging significance of TEX101 prompts us to confirm its precise expression profile. However, previous studies on the distribution of TEX101 mainly analyzed its mRNA level [11, 14, 15]. To date, systematic analysis of TEX101 protein expression has rarely been reported. In the present study, we thoroughly analyzed the expression features of TEX101 in human tissue sections. We hope that the data from our systematic analysis provide a valuable reference for understanding the functions of TEX101.

## Results and discussion

### Limited distribution of TEX101 in testis

**Figure 1:**
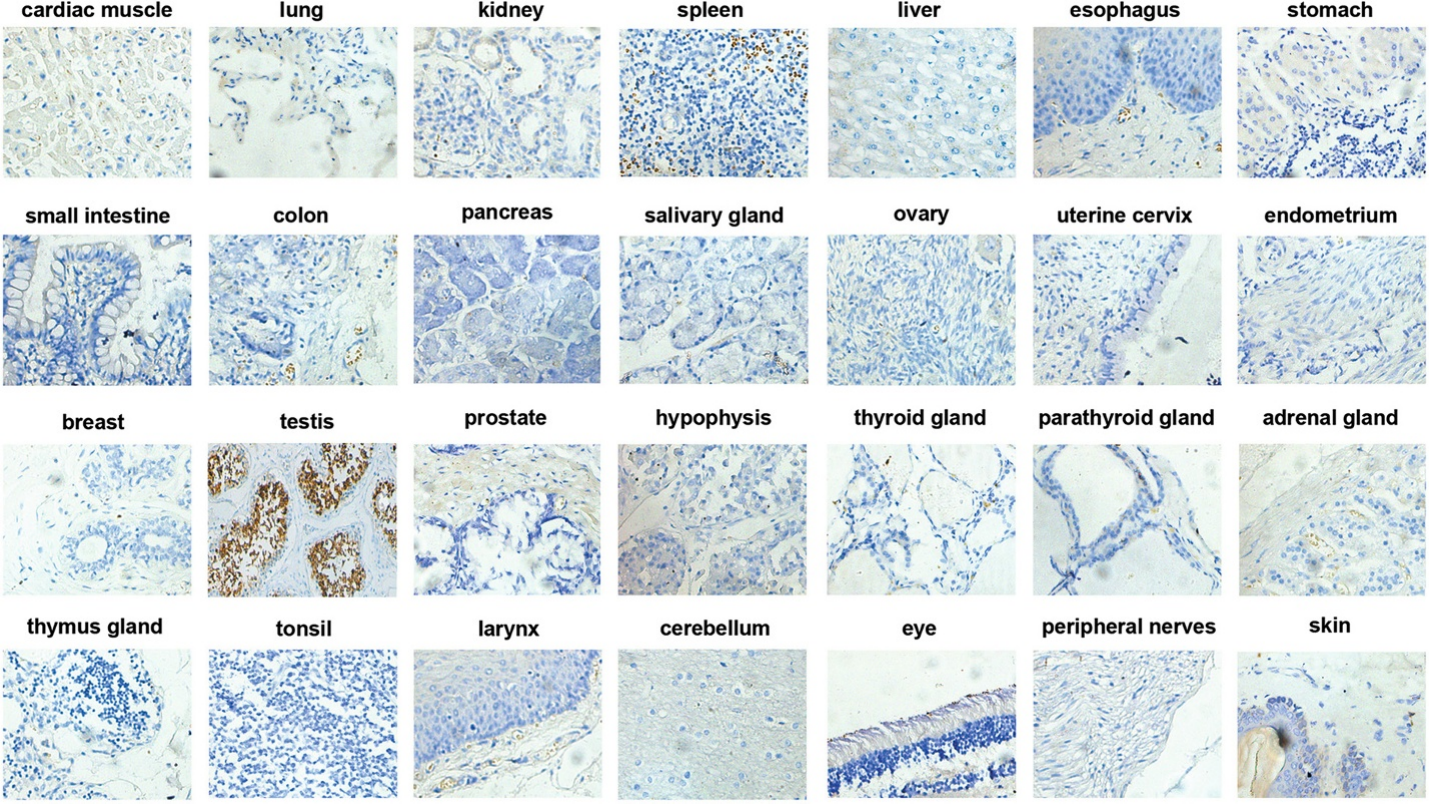
**Expression analysis of TEX101 in various human tissues.** Immunohistochemistry was performed to examine 28 types of tissues using a rabbit polyclonal antibody against TEX101. These organs covered a wide range, including cardiac muscle, lung, kidney, spleen, liver, esophagus, stomach, small intestine, colon, pancreas, salivary gland, ovary, uterine cervix, endometrium, breast, testis, prostate, hypophysis, thyroid gland, parathyroid gland, adrenal gland, thymus gland, tonsil, larynx, cerebellum, eye, peripheral nerves, and skin. The tissues sections were visualized using diaminobenzidine, and images were captured with a Leica DM2500 microscope (200×). Clear and strong staining was observed in the testis samples, but not in the other tissue samples, including ovary.

To elucidate the expression profile of TEX101, 28 types of normal human organs were collected and subjected to immunohistochemistry with a high-quality anti-TEX101 antibody. Analysis of the sections showed that testis was the only organ in which TEX101 was highly expressed (Figure 1). The staining for TEX101 was not detected in the other 27 types of organs, including ovary (Figure 1). These results confirmed that TEX101 was a testis-abundant protein. This marker has been utilized to diagnose male infertility [16, 17].

### Expression characteristics of TEX101 in testis

**Figure 2:**
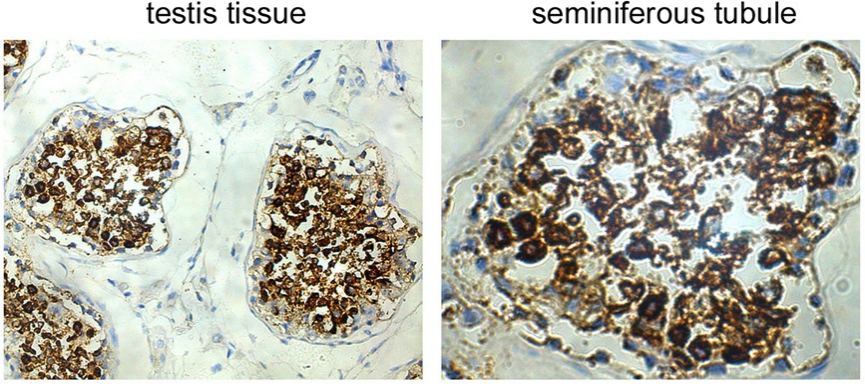
**Expression characteristics of TEX101 in testis.** The tissue localization of TEX101 in testis was investigated through immunohistochemistry. Strong staining of TEX101 was found in seminiferous tubules, but not in the connective tissue of testis (50×). In seminiferous tubules, TEX101 was highly expressed in spermatocytes and spermatids, but not in spermatogonia (400×).

Further detailed analysis revealed the expression characteristics of TEX101 in testis. The high magnification view showed that the connective tissue beyond the seminiferous tubules presented negative staining for TEX101. Although TEX101 presented very strong staining in the seminiferous tubules, the negative staining was also observed in some types of cells (Figure 2). Analysis of the sections showed that TEX101 was highly expressed in spermatocytes and spermatids but relatively expressed at low levels in spermatogonia that were near the outer edge of the seminiferous tubules (Figure 2). It is known that the maturation of male germ cells follows the course from spermatogonia to spermatocytes and finally spermatids. Our finding confirmed TEX101 should be related to the maturation process of germ cells and be considered a biomarker for the later stage of development.

### Western blotting analysis of TEX101 in testis

**Figure 3:**
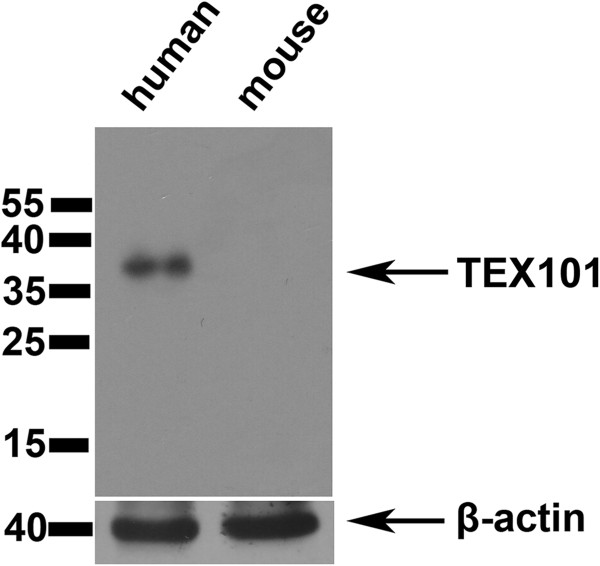
**Western blotting analysis of TEX101 in testis.** TEX101 expression in human and mouse testis was examined by Western blotting. In human testis tissues, a single, specific band of 38 kDa was detected, while no band appeared at 21 kDa. In mouse testis, no band was detected under the same experimental conditions.

Western blotting was also performed to analyze the expression features of TEX101. The tissue proteins from three different individuals were subjected to Western blotting and showed accordant features. For human testis tissues, a single, specific band of 38 kDa was detected by a polyclonal antibody of anti-TEX101 (Figure 3). However, no band was detected in mouse testis examined under the same experimental conditions (Figure 3). It is interesting that no band appeared at 21 kDa, which is the predicted molecular weight of the mature TEX101 protein. These results disclosed the actual molecular weight of TEX101 in human testis tissues and confirmed the human specificity of this TEX101 antibody.

Structure prediction showed that human and mouse TEX101 both possess a UPAR/Ly6 domain. However, only 60% homology was found in the amino acid sequences of the two proteins. This relatively low homology suggested that there was a greater evolutionary variation and there could be different functions between human and mouse TEX101. The actual molecular weight of TEX101 was determined to be 38 kDa, which is much larger than its predicted molecular weight, and this difference in size could result from post-translational modification [18, 19]. Bioinformatics analysis showed that the human TEX101 protein contains one GPI anchor site (Asn-222) and two glycosylation sites (Asn-45 and Asn-159). These modifications could lead to variations in structure and electrical charge, which might also contribute to the increased mobility of TEX101 on the gel [20, 21].

### Disappearance of TEX101 in testicular cancer

**Figure 4:**
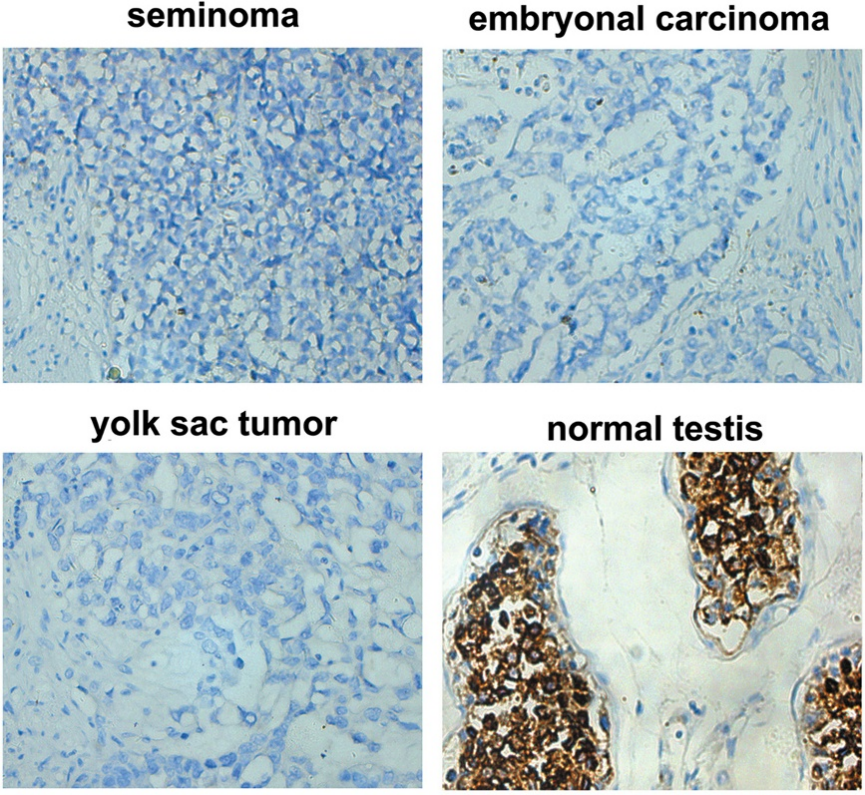
**Expression analysis of TEX101 in testicular cancer.** TEX101 expression in seminoma, yolk sac tumor, embryonal carcinoma, and normal testis tissue were examined using immunohistochemistry. The sections showed no staining of TEX101; however, all collected normal testis tissues possessed strong staining of TEX101, resulting in 100% positive expression.

Because high expression of TEX101 was detected in testis, we also wanted to know if its expression changed in testicular cancer. Thirty-eight testicular cancer tissues, including 36 samples of seminoma and two samples of yolk sac tumor and embryonal carcinoma, were subjected to immunohistochemistry. The sections showed that none of the testicular cancer tissues showed staining for TEX101, resulting in 100% negative expression (Figure 4). In contrast, all 10 samples of normal testis tissues that were collected in this study were confirmed to possess strong staining of TEX101, resulting in 100% positive expression (Figure 4).

Previous studies demonstrated that TEX101 could reduce the activities of uPA, MMPs and cathepsin B, leading to the suppression of cell growth and migration [12]. We speculated that the loss of TEX101 could disrupt the metabolic balance of the above-mentioned enzymes in the germinal epithelium, which could contribute to the growth and migration of mutant cancer cells. Therefore, our research not only found a potential biomarker for the diagnosis of testicular cancer but also provided a potential molecular mechanism for the progression of testicular cancer. However, for testicular cancer in men who do not have a germ cell line (e.g. Sertoli cell only syndrome), TEX101 should not be a good marker. Other studies found the expression of TEX101 in head and neck squamous cell carcinoma (HNSCC) as well as in nasopharyngeal carcinoma and chronic myeloid leukemia [10–12], which indicated that TEX101 could be related to cancer progression.

## Conclusions

In summary, the differential expression of TEX101 that was revealed in this study provided a valuable reference for understanding the biological functions of TEX101. The loss of TEX101 expression in seminoma also suggested its potential role in the progression of testicular cancer.

## Methods

### Materials

The primary antibodies were purchased from Atlas (rabbit anti-TEX101, R38320) and Santa Cruz (mouse anti-beta-actin, sc-81178). The secondary antibodies, including goat anti-rabbit IgG (ZB-2301) and goat anti-mouse IgG (ZB-2305), were purchased from ZSGB-Bio. The Immobile Western chemiluminescent HRP substrate (WBKLS01000) was purchased from Millipore. The antibodies used for immunohistochemistry (biotinylated anti-rabbit IgG, ZB-2010) and horseradish peroxidase-labeled streptavidin (ZB-2404) were purchased from ZSGB-Bio.

### Animal and human tissue samples

Three BALB/c mice were obtained from the Animal Center of Sichuan University. A total of 38 samples of testicular cancer tissue, 10 samples of normal testis tissue, and different organs samples from 3 different human individuals were from West China Hospital of Sichuan University. Collected tissues samples were numbered and fixed in formalin (4%) for 3 days, and the fixed samples were then washed with water for 2 h, and dehydrated with a graded ethanol series (75%, 85%, 95% and 100%) for 1 h each. Then, the samples were infiltrated with xylene for 1 h and embedded in paraffin blocks. The tissue was cut into 4 μm-thick sections, which were then placed on highly adhesive slides. The sections were stored at 4°C until the subsequent experiments were performed. This study was approved by the institutional ethics committee of Sichuan University. All of the patients assented to participate in the study after providing informed consent.

### Immunohistochemistry

Immunohistochemistry was performed using the SP (streptavidin-peroxidase) method. Briefly, the sections were pretreated with 10 mM citrate buffer (pH 6.0), incubated with 3% H_2_O_2_ for 10 min, and blocked in 5% BSA for 10 min at room temperature. The sections were then probed with the primary antibody against TEX101 (1:300, diluted with PBS) overnight at 4°C. Subsequently, the sections were washed with PBS three times and incubated with biotinylated anti-rabbit IgG for 30 min at 37°C. After washing three times, the sections were incubated with horseradish peroxidase-labeled streptavidin for 30 min at 37°C and then washed four times with PBS. Finally, the sections were visualized using diaminobenzidine (DAB). Images were captured (200× and 400×) using high-resolution microscopy (Leica DM2500 microscope) and analyzed using Leica Application Suite software.

### Western blotting

The collected tissue samples of human and mouse testis were ground in liquid nitrogen, lysed in RIPA Buffer for 30 min on ice, and centrifuged at 16,000 g for 30 min at 4°C. After measurement using a Protein Assay kit (Bio-Rad), the proteins were separated on 12% SDS-PAGE gels and transferred to PVDF membranes (Bio-Rad, 162–0177). The membranes were blocked in TBST (20 mM Tris (pH 7.5), 150 mM NaCl, and 0.1% Tween 20) that contained 5% skimmed milk for 3 h at 37°C and then incubated with the primary antibodies against TEX101 (1:1000, diluted by TBST) for 2 h at 37°C. Subsequently, the membranes were washed three times with TBST and probed with secondary antibodies conjugated to HRP (1:15000, diluted by TBST) for 1 h at 37°C. After washing three times, the membranes were developed using the Immobilon Western kit (chemiluminescent HRP substrate) (Millipore).
